# Oxytocin during Development: Possible Organizational Effects on Behavior

**DOI:** 10.3389/fendo.2015.00076

**Published:** 2015-05-19

**Authors:** Travis V. Miller, Heather K. Caldwell

**Affiliations:** ^1^Laboratory of Neuroendocrinology and Behavior, Department of Biological Sciences, Kent State University, Kent, OH, USA; ^2^School of Biomedical Sciences, Kent State University, Kent, OH, USA

**Keywords:** affiliative behavior, aggressive behavior, estrogen receptor alpha, maternal behavior, oxytocin receptor, parental behavior, sexual behavior, vasopressin

## Abstract

Oxytocin (Oxt) is a neurohormone known for its physiological roles associated with lactation and parturition in mammals. Oxt can also profoundly influence mammalian social behaviors such as affiliative, parental, and aggressive behaviors. While the acute effects of Oxt signaling on adult behavior have been heavily researched in many species, including humans, the developmental effects of Oxt on the brain and behavior are just beginning to be explored. There is evidence that Oxt in early postnatal and peripubertal development, and perhaps during prenatal life, affects adult behavior by altering neural structure and function. However, the specific mechanisms by which this occurs remain unknown. Thus, this review will detail what is known about how developmental Oxt impacts behavior as well as explore the specific neurochemicals and neural substrates that are important to these behaviors.

## Introduction

In the landmark paper by Phoenix et al. (1), the organizational effects of gonadal steroids on sexual behavior in guinea pigs (*Cavia porcellus*) were established and resulted in the formulation of the “organizational/activational hypothesis of sexual differentiation.” This hypothesis, which states that perinatal exposure to gonadal steroids is important for the sexual differentiation of the brain and behavior, is part of the foundation on which the field of behavioral neuroendocrinology has been built. Given the importance of this concept, it is perhaps not surprising that in the last 55 years this hypothesis has been extended to include another critical time period – puberty – as well as other hormones and behaviors, with one of these hormones being oxytocin (Oxt) (2–4).

Oxytocin is a mammalian neurohormone composed of nine amino acids, known for its peripheral effects on parturition and lactation (5–7), as well as its central neuromodulatory effects on social behaviors such as affiliative, aggressive, and parental behaviors (8, 9). While much of the work on Oxt has focused on its involvement in the acute modulation of behavior, there is also evidence that exposure to Oxt during early life is important for the proper development of neural pathways and subsequent sex-specific behaviors. These latter observations have led researchers to hypothesize that Oxt has organizational effects on the brain (3, 10). This is an exciting possibility as developmental exposure to Oxt appears to impact many of the species-specific and sex-specific social behaviors it is known to modulate in adulthood – thus, a reevaluation and broadening of our understanding of Oxt’s effects seems warranted. To encourage this shift in paradigm, in this review we will highlight recent research on how developmental exposure to Oxt affects behavior as well as the specific neurochemicals and neural substrates that underlie these behaviors.

## Oxytocin’s Postnatal and Peripubertal Effects on Behavior

Noonan and colleagues (10) were the first to hypothesize that Oxt might have organizational effects on the brain and behavior. Their work demonstrated that a single intracisternal injection of Oxt in postnatal day (PND) 3 rats (*Rattus norvegicus*) significantly increases novelty-induced grooming at 4 months of age (10) [grooming is known to be directly enhanced by Oxt administration in adults (11)]. However, when a study by Boer and colleagues failed to replicate the aforementioned findings in an open-field test (12), investigations into the organizational effects of Oxt largely fell by the wayside. Yet, in the last 12 years, there has been a resurgence of research in this area, with studies in numerous species providing converging evidence that Oxt during development can have permanent effects on the brain and behavior (3, 13–29) (see Table 1). The implications are profound, as they are changing the way that we think about how Oxt works – no longer as just a neuromodulator, but rather as a neurohormone that contributes to the development of behaviors that are essential for survival.

**Table 1:** **A summary of the design and findings of studies investigating how developmental manipulation of oxytocin activity affects long-term behavioral expression**.

Species	Treatment	Age at treatment	Age at assay	Behavioral outcomes in females	Behavioral outcomes in males	Reference
Prairie vole	3.0 μg Oxt i.p.	PND0	PND60–90	ND	Rescued social behavior diminished by saline, ↑ partner preference	(19)
Prairie vole	0.3 μg OTA i.p.	PND0	PND60–90	ND	Rescued social behavior diminished by saline	(19)
Prairie vole	3.0 μg Oxt i.p.	PND0	Adult	↑ Aggression, ↓ social behavior after exposure to male	↔	(18)
Prairie vole	0.3 μg OTA i.p.	PND0	Adult	↔	↔	(18)
Prairie vole	3.0 μg Oxt i.p.	PND0	PND8	↔	↔	(23)
Prairie vole	0.3 μg OTA i.p.	PND0	PND8	↓ Ultrasonic vocalizations after isolation	↔	(23)
Prairie vole	3.0 μg Oxt i.p.	PND0–7	PND8	↔	↔	(23)
Prairie vole	0.3 μg OTA i.p.	PND0–7	PND8	↑ Ultrasonic vocalizations after isolation	↔	(23)
Prairie vole	3.0 μg Oxt i.p.	PND0	PND21	↔	↔	(14)
Prairie vole	0.3 μg OTA i.p.	PND0	PND21	↔	↓ Parental behavior, ↑ pup-directed aggression	(14)
Prairie vole	3.0 μg Oxt i.p.	PND0	PND60	↔	↔	(14)
Prairie vole	0.3 μg OTA i.p.	PND0	PND60	↔	↔	(14)
Prairie vole	3.0 μg Oxt i.p.	PND0	PND75	↓ Mating bout frequency	ND	(20)
Prairie vole	0.3 μg OTA i.p.	PND0	PND75	↓ Mating bout frequency, ↑ litter production success	ND	(20)
Prairie vole	1.0 mg/kg Oxt i.p.	PND0	PND55–69	↔	ND	(16)
Prairie vole	2.0 mg/kg Oxt i.p.	PND0	PND55–69	↔	ND	(16)
Prairie vole	4.0 mg/kg Oxt i.p.	PND0	PND55–69	↑ Pup retrievals	ND	(16)
Prairie vole	8.0 mg/kg Oxt i.p.	PND0	PND55–69	↑ Preference for stranger	ND	(16)
Prairie vole	750 nL CMV-Oxtr NAcc-specific	PND21	PND60–88	↑ Parental behavior, ↑ preference for partner	ND	(57)
Prairie vole	0.08 IU/kg Oxt intranasally	PND21–42	PND43–60	↔	↑ Preference for stranger	(43)
Prairie vole	0.80 IU/kg Oxt intranasally	PND21–42	PND43–60	↔	↑ Preference for stranger	(43)
Prairie vole	8.00 IU/kg Oxt intranasally	PND21–42	PND43–60	↔	↔	(43)
Mandarin vole	3.0 μg Oxt s.c.	PND0	PND60–90	↑ Aggression after exposure to male	↑ Social contact	(35)
Mandarin vole	3.0 μg Oxt s.c.	PND0	PND60–90	↑ Preference for partner, suppressed maintenance of preference, ↓ aggression toward stranger	↑ Mounting of partner, ↓ aggression toward stranger	(37)
Rat	1.0 μg/2.0 μL Oxt intracisternally	PND3–4	PND120	↑ Novelty-induced grooming	↑ Novelty-induced grooming	(10)
Rat	1.0 mg/kg Oxt s.c.	PND10–14	PND60–94	↑ Weight gain, ↑ tail-flick withdrawal latency	↑ Weight gain, ↑ tail-flick withdrawal latency	(63)
Rat	1.0 mg/kg Oxt i.p., 0.5 μg EB	PND0–7	PND75	↓ Sexual receptivity	ND	(28)
Rat	1.0 mg/kg Oxt i.p., 5.0 μg EB	PND0–7	PND75	↓ Sexual receptivity	ND	(28)
Rat	1.0 mg/kg Oxt i.p., 10.0 μg EB	PND0–7	PND75	↔	ND	(28)
Rat	0.1 mg/kg OTA i.p., 0.5 μg EB	PND0–7	PND75	↓ Sexual receptivity	ND	(28)
Rat	0.1 mg/kg OTA i.p., 5.0 μg EB	PND0–7	PND75	↔	ND	(28)
Rat	0.1 mg/kg OTA i.p., 10.0 μg EB	PND0–7	PND75	↔	ND	(28)
Rat	1.0 mg/kg Oxt i.p.	PND33–42	PND50–72	ND	↑ Open-field exploration, ↑ social interaction, ↓ ethanol consumption	(41)
Rat	0.5 mg/kg Oxt i.p.	PND28–55	PND70–72	ND	↑ Social proximity	(42)
Rat	1.0 mg/kg Oxt i.p.	PND28–55	PND70–72	ND	↑ Social proximity	(42)
Rat	0.5 mg/kg TGOT i.p.	PND28–55	PND70–72	ND	↔	(42)
Rat	1.0 mg/kg TGOT i.p.	PND28–55	PND70–72	ND	↔	(42)
Mouse	2.0 μg Oxt s.c.	PND0	PND1–3	Rescued feeding behavior in Magel2^−/−^ mice	Rescued feeding behavior in Magel2^−/−^ mice	(64)
Mouse	3.0 μg OTA s.c.	PND0	PND1–3	Lethal feeding deficiency	Lethal feeding deficiency	(64)
Mouse	3.0 μg Oxt i.p.	PND0	8–15 weeks	↔	↔	(29)
Mouse	0.3 μg Oxt i.p.	PND0	8–15 weeks	↔	↔	(29)
Mouse	3.0 μg OTA i.p.	PND0	8–15 weeks	↓ Parental care	↓ Parental care	(29)
Mouse	0.3 μg OTA i.p.	PND0	8–15 weeks	↔	↔	(29)
Mouse	0.15 IU Oxt intranasally	12–23 weeks	1 h post	ND	↓ Social behavior	(45)
Mouse	0.30 IU Oxt intranasally	12–23 weeks	1 h post	ND	↓ Social behavior	(45)
Mouse	0.80 IU Oxt intranasally	PND21–50	PND55	Rescued diminished social sniffing in BTBR mouse	↔	(44)
Pig	50.0 μg Oxt intranasally	PND1–3	2–8 weeks	↑ Aggression	↑ Aggression	(39)

*ND, no data; EB, estradiol benzoate*.

### Sexual, affiliative, and aggressive behaviors

Work in prairie voles (*Microtus ochrogaster*) has shown that there are clear behavioral consequences when Oxt is manipulated during early postnatal development, and that these effects are sex specific; this latter observation is perhaps not surprising since it is well known that Oxt expression is sexually dimorphic in numerous species, including prairie voles (30–34). In female prairie voles, a single injection of Oxt on PND1 results in increases in intrasexual aggression in adults, suggesting strengthened pair bond formation (18), whereas an injection of both Oxt and an Oxt receptor antagonist (OTA), also on PND1, decreases the frequency of mating bouts in both adult males and females (20). A single postnatal injection of Oxt also increases intrasexual aggression in adult female mandarin voles (*Lasiopodomys mandarinus*) after exposure to a male (35). In male prairie voles, an injection of Oxt at both low (1 mg/kg) and high (4 mg/kg) doses on PND0 increases partner preference and social contact in adults compared to controls (19, 36); however, a dose of 2 mg/kg does not (36). Male mandarin voles that receive a single postnatal injection of Oxt increase their mounting behavior at PND60 (37). So, at least in voles, developmental Oxt appears to increase pair bond formation in females, and increase affiliative behaviors in males, whereas the effects on sexual behavior appear to be stimulatory in both males and females; though they may be dose dependent in a manner that is not linear.

In mice (*Mus musculus*), there are sex differences in the effects of neonatal Oxt manipulation on affiliative behaviors, though the findings differ from observations in prairie voles. Female mice administered an OTA (3 μg/20 μL) on the day of birth have decreases in social approach when tested at 8–15 weeks (29) as measured in a three chambered apparatus based on that developed by Crawley (38). On the other hand, male mice administered an OTA on PND0 display social approach behaviors similar to what is observed in the control conditions (29). So, in mice it appears that Oxt exposure in neonate females may promote affiliative behaviors, while having no effect in males. Exploration of why there may be no effect in male mice can be found in the section titled “Potential Effects of Oxytocin During Fetal Development.”

The aforementioned impact of developmental Oxt on behavior is not limited to rodents. Postnatal intranasal Oxt administration in 2.5- to 8-week-old pigs (*Sus scrofa*) increases intrasexual aggressive behavior and decreases social contact (39), which is similar to what is observed in female prairie voles (18). [Pigs were selected as an experimental model because their neuroanatomy, as well as their physiology and development, is more similar to humans than rodent models (40).] This particular study is unusual in that it is the only one to use an ungulate animal model, as well as one of only a few studies to use intranasal Oxt administration. Because of the uniqueness of this species, it is not known if these findings are broadly applicable to other species.

There is also evidence that Oxt’s developmental effects on sociability may not be limited to the perinatal period, but may also extend into peripubertal development (2). Bowen and colleagues administered daily Oxt injections to male rats from PND33 to PND42 and then tested them in a social interaction test on PND55. Oxt-treated males spent more time in close proximity to conspecifics and made more active social contacts than controls (41). Researchers from this same research group later conducted an experiment in which male rats were given Oxt injections every 3 days from PND28 to PND55. When tested in a social interaction test at PND70, their behavior appeared similar to what was described above, with their spending more time in close proximity to conspecifics than control animals (42). There have also been studies investigating the effects of peripubertal intranasal Oxt on behavior in rodents. Male prairie voles administered low, medium, and high doses of intranasal Oxt daily during the approximate time between weaning and sexual maturity, from PND21 until 42, display increases in social contact during the treatment window, and those given low and medium doses increase their preference for strangers when tested from PND43–60 (43). In female BTBR mice (a model of autism spectrum disorders), daily intranasal Oxt treatment from PND21 to 50, followed by testing on PND55, increases time spent sniffing a novel mouse over a novel object, essentially rescuing “sociability” to levels observed in wild-type mice (44). Another study found that chronic intranasal Oxt from 12 to 23 weeks reduced social behavior in adult male mice when they were tested 1 h after Oxt administration (45). While the studies described above may differ somewhat in terms of the details of their findings, it does appear that intranasal Oxt treatment during peripubertal development facilitates social interactions in both males and females. However, it is not clear how long lasting these effects are. Given that the peripubertal period is another critical developmental window for the organizational effects of hormones (2), more work focused on these types of questions is needed, especially as intranasal Oxt is being considered as a treatment for various neurodevelopmental conditions (46–48), in particular children and adolescents diagnosed with autism spectrum disorders (49–52).

### Parental behavior

In male prairie voles, treatment with an OTA in neonates decreases alloparental behaviors. Specifically, males injected with an OTA within 24 h of birth and later tested on PND21 decrease their parental care, as measured by fewer retrievals, less time spent huddling over pups, and increases in pup-directed attacks. These effects also appear to be transient, as they are not observed when the same animals are tested again on PND60 (14). In laboratory mice, treatment with an OTA also reduces alloparental care, but in both sexes. Specifically, treatment with an OTA on PND0 decreases the total number of pups retrieved by females and increases pup retrieval latencies in males (29). Oxt treatment also increases the responsiveness of females to pups, as measured by approach times, although this effect is dose dependent, with the lowest dose of Oxt resulting in longer approach times compared to saline controls (16). Thus, it appears that Oxt signaling is important for normal displays of alloparental care, which is consistent with its role in lowering the threshold for maternal care in rodents (53–56).

Work by Keebaugh and Young has utilized viral vectors to overexpress the Oxtr and study the organizational effects of Oxt on behavior during puberty. By injecting an adeno-associated viral vector into the nucleus accumbens (NAcc) shell of female prairie voles at PND21 they were able to facilitate alloparental care, as measured by reductions in approach times and increases in time spent licking and grooming pups compared to controls (57). This gene delivery approach has helped to identify a specific neural substrate – the NAcc – on which Oxt may act. The NAcc, which is a part of the brain’s reward circuit, is known to be important in numerous motivated behaviors – it not only expresses the Oxtr but is also one of the regions in which the Oxtr is more highly expressed in biparental prairie voles compared to non-monogamous species that do not exhibit biparental care (58–62).

### Other behaviors

While only a few studies have investigated Oxt’s organizational effects on other behaviors, these studies confirm that Oxt’s developmental effects are not limited to its impact on social behavior. In rats, repeated administration of Oxt between PND10 and PND14 results in weight gain in both males and females, increases in the gut hormone cholecystokinin, and longer withdrawal latencies in the tail-flick test at PND60, which suggests an increase in pain threshold (63). Male and female mice with a genetic disruption that models Prader–Willi syndrome usually exhibit a lethal feeding deficiency. This feeding deficiency can be rescued with an injection of Oxt on PND0, or induced in wild-type mice by injecting an OTA 1–1.5 h after birth (64). These observations are consistent with what is observed in Oxt and Oxtr knockout (−/−) mice. These mice develop late-onset obesity in the absence of hyperphagia (65, 66). However, these developmental effects of Oxt differ from what is observed in adults, where Oxt is hypothesized to have anorexigenic effects (67). Since developmental Oxt appears to impact aspects of energy homeostasis, which in turn can affect behavior, additional work in this area is warranted.

Finally, in the study by Bowen and colleagues (41) mentioned in the previous section, peripubertal Oxt administration also affects anxiety-like behavior and ethanol consumption later in life. Male rats given daily Oxt injections from PND33 to PND42 and tested in an emergence test on PND50 traveled further and spent more time in the open-field compared to controls, which would suggest that Oxt had an anxiolytic effect. When tested on PND72 and later, Oxt-treated males consumed significantly less alcohol (i.e., beer) than controls, while water consumption remained unaffected (41).

### Summary

It is apparent that Oxt in early postnatal development and during puberty can result in long-lasting changes in behavior, including behaviors that have traditionally been associated with Oxt’s acute neuromodulatory effects, such as affiliative and sexual behaviors, as well as non-social behaviors, such as nociception and ethanol consumption. While there is a lack of consensus in the data, this is in part a reflection of the difficulty of these types of studies, as there are many experimental possibilities that could result in very different outcomes. Particularly crucial is the timing window for Oxt administration, the dose of Oxt, and the behavioral endpoints measured. It is also important to note that Oxt’s organizational effects appear to be sex- and species-specific, which is consistent with the complexity of its neuromodulatory role in adulthood. Thus, future behavioral work will need to continue to take a broad approach to identify Oxt’s potential organizational effects, as some of these behavioral changes, such as energy metabolism and anxiety, could affect a variety of other behaviors. In the meantime, rigorous investigation of Oxt’s organizational effects on neural structure and function may help clarify mechanisms by which Oxt affects brain development and ultimately behavior.

## Oxytocin’s Developmental Effects on Neurochemicals and Neural Substrates

The behavioral effects of postnatal and peripubertal Oxt must be rooted in structural and functional modifications to neurons, such as changes in gene expression, axonal guidance, or cell morphology. Therefore, in this section, we review what is known about how Oxt may be impacting these systems.

### Effects on estrogen receptor alpha

It is well established that gonadal steroids play a significant role in regulating Oxt activity (68–71). While androgens and progesterone modulate Oxt and Oxtr expression (72–75), it is the estrogens that seem to have the greatest impact on the Oxt system (68, 70, 74, 76–78). Further, these effects are not unidirectional, with the Oxt system altering gonadal steroid systems, specifically the expression of estrogen receptor α (ERα). Neonatal injections of Oxt increase ERα expression in the ventromedial hypothalamus (VMH) of adult females and Oxt treatment on PND0 increases the number of ERα-immunoreactive (ERα-ir) cells in the VMH of 3-week-old prairie voles (26). These effects appear to be rapid since neonatal prairie voles treated with Oxt also have increases in ERα-ir (24, 79) and ERα mRNA expressions (79). Similar to prairie voles, rats that are repeatedly administered Oxt from PND0 until PND7 have increases in ERα-ir at PND75 (28) and neonatal Oxt manipulation increases the expression of ERα in the hippocampus (79), ventral lateral septum (LS), and central nucleus of the amygdala (24). These results differ from reports in females where a single injection of an OTA on PND1 decreases expression of ERα in the medial preoptic area (MPOA) of adult female prairie voles, as well as increases ERα expression in the BNST, and possibly decreases expression in the medial amygdala (MeA) (26) and repeated administration of an OTA from PND0 to PND7 decreases the expression of ERα in the MPOA of adult female rats (28).

### Effects on the oxytocin and vasopressin systems

Early exposure to relevant stimuli is known to permanently alter the responsiveness of a hormone receptor; this phenomenon is known as *hormonal imprinting* (80). Based on this observation, it is reasonable to suspect that early Oxt exposure could affect the development of the Oxt system itself. Data from several species clearly support this idea; however, the findings are not consistent between sexes and species – in keeping with Oxt’s known intersexual and interspecific variation. In female prairie voles, neonates treated with Oxt and an OTA have increases in Oxt immunoreactivity (Oxt-ir) within the PVN by 3 weeks of age. Yet, in males, Oxt has no effect on Oxt-ir, but treatment with an OTA does decrease arginine vasopressin (Avp) immunoreactivity in the PVN (27).

In addition to changes in peptide expression, neonatal manipulation of Oxt impacts Avp 1a receptor (Avpr1a) binding in a sexually dimorphic manner. Specifically, in female prairie voles, Oxt treatment on PND0 decreases Avpr1a binding in the MPOA, BNST, LS, cingulate cortex (CgCtx), and medial thalamus on PND60, but in males it increases Avpr1a binding in the CgCtx. OTA treatment in females decreases Avpr1a binding only in the BNST and CgCtx, while in males it decreases Avpr1a binding in the MPOA, BNST, and LS (15). Taken together, the delivery of Oxt or an OTA during early postnatal life appears to have nearly opposite effects on adult Avpr1a expression in females and males. Similar to what was discussed in terms of behavioral effects, there does appear to be a “critical period” of organization that extends into peripubertal development, with peripubertal Oxt administration increasing Oxtr mRNA expression in the hypothalamus (41) and plasma Oxt levels (42) in adult male rats.

### Other effects

While studies on the organizational effects of Oxt on other neurochemical systems are few and far between, there is evidence that perinatal exposure to Oxt can influence the functioning of the stress axis. Female prairie voles administered Oxt on PND1 have reductions in baseline plasma corticosterone by PND8 compared to animals treated with saline or an OTA (23). In neonatal pigs, repeated Oxt treatment increases adrenocorticotropic hormone (ACTH) at 8 weeks of age and decreases responsiveness in the dexamethasone suppression test at 11 weeks; indicative of dysregulation of the glucocorticoid response. However, pigs that receive Oxt have less blunting of the cortisol response than controls (39). So, not only is Oxt able to acutely regulate stress responses (81, 82) but appears to also be involved in the development of long-term responsiveness to stress.

There are also reported effects of neonatal Oxt on adrenergic and serotonergic receptors, which may contribute to the aforementioned effects of Oxt on feeding and social behaviors (see Other Behaviors). In rats, chronic neonatal Oxt treatment alters α_2_ adrenergic receptor (α_2_r) kinetics in PND130 male rats; these changes are dependent upon the nutrition of the dam to which the pups were born. Specifically, Oxt treatment rescues the affinity (described by the dissociation constant *K*_d_), which is decreased in placebo treated neonates of food-restricted dams compared to offspring of dams fed *ad libitum*, of the α_2_r for its ligand within the nucleus of the solitary tract (NTS) and increases the number of α_2_r binding sites (described by *B*_max_) in the hypothalamus and amygdala of offspring born to food-restricted dams compared to controls. In pups born to dams fed *ad libitum* Oxt treatment decreases the affinity of the α_2_r for its ligand in the hypothalamus, and increases the number of binding sites in the hypothalamus and NTS (83). In male prairie voles, neonatal Oxt administration also results in increases in serotonergic axon density in the anterior hypothalamus, cortical amygdala, and VMH at PND21 (21), as well as decreases dopamine turnover in the hypothalamus as well as serotonin turnover in the hypothalamus, medulla oblongata, and striatum of 4-month-old female rats (84). Thus, not only does developmental Oxt exposure affect many different neural circuits, these effects can also be highly dependent on the state of the dam. This is in keeping with a large body of research showing that maternal physiology, particularly stress (85–87) and nutrition (88–90), can greatly impact the neural and behavioral development of offspring. The previously described studies have opened the door to exploring what role developmental Oxt might play in these effects.

### Summary

**Figure 1:**
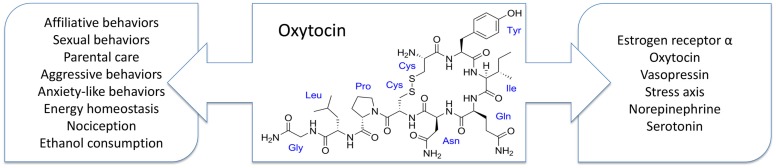
**Developmental exposure to oxytocin is known to affect many behaviors (left) and neurotransmitter and neurohormone systems (right)**. These behavioral effects are often species and sex specific, which is consistent with oxytocin’s neuromodulatory role in adults. However, how these behavioral changes are rooted in the observed alterations in neurochemistry remains unknown.

The organizational effects of Oxt extend to many different neurochemical systems including the gonadal steroids, the Oxt and Avp systems, and the stress axis, which suggests that Oxt’s effects are widespread and complex (Figure 1). The brain regions most commonly affected by developmental Oxt exposure are the VMH, MPOA, BNST, LS, PVN, and several nuclei of the amygdala. Oxt or the Oxtr is expressed in some of these regions (68, 91), though their expression in these brain areas often varies depending on species, age, and sex. What is particularly interesting about the aforementioned brain nuclei is that many of them are a part of the “social behavioral network,” which is comprised of neuroanatomical areas or “nodes” that are interconnected, express gonadal hormone receptors, appear to be influenced by Oxt, and are important in the regulation of many types of social behaviors (92, 93). Work from Bruce Cushing’s laboratory suggests that the BNST and MeA are particularly important for the developmental effects of Oxt and ERα on social behavior (24, 26, 94) but further investigation into the entire social behavioral network is needed.

## Potential Effects of Oxytocin during Fetal Development

While research into the neonatal and peripubertal developmental effects of Oxt has been ever increasing, an area that remains largely unexplored is the potential for Oxt to have organizational effects during embryonic development. These potential effects are relevant, in part, because of the increased use of Oxtr agonists, such as Pitocin, and antagonists, such as Atosiban, during human pregnancy in order to manage labor timing (95, 96). While the developmental consequences of these interventions have been reviewed elsewhere (95), the possible behavioral implications of exposing human fetuses to exogenous Oxt or Oxtr antagonists should not be ignored. Because of the complexity and ethical considerations in humans our best hope for elucidating the role of fetal Oxt is the use of animal models.

In mice, there is evidence that *in utero* exposure to Oxt is important for normal intermale aggressive behavior in adulthood. Specifically, male Oxt^−/−^ mice that are born to null mutant dams show heightened aggressive behavior in adulthood (97, 98). While this phenotype cannot be rescued if the pups are cross-fostered to wild-type dams (99), it is not observed when male mice are born to heterozygous dams. One of the key differences between pups born to null mutant dams versus those born to heterozygous dams is the absence or presence of maternal Oxt. The hypothesis that the maternal Oxt may be signaling in the fetal brain is supported by studies using male Oxtr knockout (Oxtr^−/−^) mice, which lack Oxtr signaling throughout development. These mice also have heightened aggressive behavior in adulthood (99, 100). However, male forebrain Oxtr knockout (Oxtr Fb/Fb) mice (101, 102), in which the *Oxtr* gene is excised 21–28 days after birth, have normal aggressive behavior in adulthood (100). These data suggest that Oxt signaling via the Oxtr during fetal development might be important for displays of aggressive behavior, and perhaps other behaviors in adulthood.

Unfortunately, very little is known about the developmental time course of the Oxt system in rodents. In rats, Oxt mRNA is observed as early as embryonic day (E) 15.5 in the PVN and E18.5 in the SON (103). The mRNA for the Oxt carrier protein neurophysin-I is available as early as E16 in the PVN and SON, and the Oxt peptide is seen by PND7 in the SON and PVN and E21 in the pituitary (104). Prairie voles also have a postnatal increase in Oxt peptide expression, with the number of Oxt expressing neurons significantly increasing from PND1 to PND21 in both males and females (27). While less is known about the development of the Oxtr, in rats, Oxtr binding has been identified as early as E14 in undifferentiated neurons (104). In mice, a study by Hammock and Levitt (105), which focused primarily on Oxtr binding during postnatal development, found Oxtr binding in the brains of E18.5 C57BL/6J mice. However, this was the only embryonic time point that was examined. It is plausible that the “critical window” for the manipulation of the Oxt system is not the same in males as in females, since there could be sex differences in its development. Therefore, future research in this area should consider the potential for sex differences in the development of the Oxt system, as it may help to inform the conclusions that are drawn from the data.

## Conclusion

Compared to what we understand about Oxt in adulthood, research into its role in development is still in its early stages and there is much to do before we really have a handle on its effects on the brain and behavior. For instance, what other behaviors are affected? How conserved are Oxt’s effects across species, or within a particular sex? How are these organizational changes grounded in alterations of neuronal structure and function? How broad is the “critical window” for Oxt’s effects? What are the implications for human offspring? Therefore, examination of the mechanisms that may underlie behavioral changes, including the identification of specific neural substrates, is of the utmost importance.

This is an exciting time in behavioral neuroendocrine research due to increased interest in Oxt and the social brain as well as great advancements in the foundational work, which has methodically examined the neuromodulatory effects of Oxt in animal models. However, there is still much we do not understand about the Oxt system, and filling this “knowledge gap” becomes more vital as the interest in using Oxt in clinical settings continues to increase. For the last several years, intranasal Oxt has been marketed as the “cuddle” or “love” hormone, and is being used in a variety of contexts as a therapeutic agent to promote prosocial behaviors in humans. Much of this work has been performed in the absence of dose response studies, without serious consideration of the potential developmental or long-term effects, and with little attention paid to where in the brain these effects might be mediated. Thus, it is perhaps not surprising that more recent studies in humans suggest that Oxt’s effects are nuanced (as the data from animal models would suggest) and that intranasal Oxt treatment can have undesirable effects (106–108). In light of this, basic research on the Oxt system becomes ever more critical, particularly since our understanding of the developmental role of Oxt is expanding. An important first step is to home in on specific circuits, focusing on studies that will shed light on the interactions between numerous brain regions and behaviors. This approach will allow scientists to elucidate the specific mechanisms of Oxt’s organizational effects on behavior – be they genetic, epigenetic, or neuroanatomical – which can then be used not only to inform human studies but also identify any conserved mechanisms between sexes and species.

## Author Contributions

Both TM and HC conceived of and drafted the work.

## Conflict of Interest Statement

The authors declare that the research was conducted in the absence of any commercial or financial relationships that could be construed as a potential conflict of interest.
